# **ASO Author Reflections**: **Refining Prognosis in Stage III Cutaneous Melanoma with Non-nodal Regional Metastases**

**DOI:** 10.1245/s10434-026-19238-4

**Published:** 2026-02-09

**Authors:** Nazia Riaz, Roger Olofsson Bagge

**Affiliations:** 1 Department of Surgery, Institute of Clinical Sciences, Sahlgrenska Academy, University of Gothenburg, Gothenburg, Sweden; 2 Department of Surgery, Sahlgrenska University Hospital, Gothenburg, Västra Götalandsregionen Sweden

## Past

Non-nodal regional metastases, including microsatellites, satellites, and in-transit metastases, are a distinctive manifestation of locoregional disease in cutaneous melanoma associated with unfavourable disease biology and poor survival.^1^ The American Joint Committee on Cancer (AJCC) 8th edition incorporates microsatellites, satellites, and in-transit metastases into “c” nodal subcategories, underscoring their adverse prognostic weight.^2^ A small proportion of patients presents with non-nodal regional metastasis but without regional nodal involvement. Whether performing a sentinel lymph node biopsy in these patients provides additional prognostic information remains unclear.

## Present

Using the large, multi-institutional Sentinel Lymph Node Working Group (SLNWG) database spanning from 1988 to 2025, our study provides real-world evidence that non-nodal regional metastases are independently associated with sentinel lymph node positivity and adverse melanoma-specific, relapse-free, and overall survival.^3^

## Future

Collectively, these findings validate the AJCC staging framework and reinforce the prognostic relevance of sentinel lymph node status in clinically node-negative patients with non-nodal regional metastases. Although sentinel lymph node assessment does not influence neoadjuvant therapy eligibility in this already stage III patient population, it does add clinically meaningful prognostic resolution. Moreover, sentinel lymph node biopsy in clinically node-negative patients with non-nodal regional metastases may still offer regional nodal basin control, particularly among patients who may not be candidates for neoadjuvant or adjuvant therapy.
